# Role of RPTPβ/ζ in neuroinflammation and microglia-neuron communication

**DOI:** 10.1038/s41598-020-76415-5

**Published:** 2020-11-20

**Authors:** Rosalía Fernández-Calle, Milagros Galán-Llario, Esther Gramage, Begoña Zapatería, Marta Vicente-Rodríguez, José M. Zapico, Beatriz de Pascual-Teresa, Ana Ramos, M. Pilar Ramos-Álvarez, María Uribarri, Marcel Ferrer-Alcón, Gonzalo Herradón

**Affiliations:** 1 Departamento de Ciencias Farmacéuticas y de La Salud, Facultad de Farmacia, Universidad San Pablo-CEU, CEU Universities, Urbanización Montepríncipe, 28925 Alcorcón, Madrid Spain; 2 Departamento de Química y Bioquímica, Facultad de Farmacia, Universidad San Pablo-CEU, CEU Universities, Urbanización Montepríncipe, 28925 Alcorcón, Madrid Spain; 3 BRAINco Biopharma, S.L., Bizkaia Technology Park, Derio, Spain

**Keywords:** Biochemistry, Neuroscience

## Abstract

Pleiotrophin (PTN) is a cytokine that is upregulated in different neuroinflammatory disorders. Using mice with transgenic PTN overexpression in the brain (*Ptn*-Tg), we have found a positive correlation between *iNos* and *Tnfα* mRNA and *Ptn* mRNA levels in the prefrontal cortex (PFC) of LPS-treated mice. PTN is an inhibitor of Receptor Protein Tyrosine Phosphatase (RPTP) β/ζ, which is mainly expressed in the central nervous system. We aimed to test if RPTPβ/ζ is involved in the modulation of neuroinflammatory responses using specific inhibitors of RPTPβ/ζ (MY10 and MY33-3). Treatment with MY10 potentiated LPS-induced microglial responses in the mouse PFC. Surprisingly, MY10 caused a decrease in LPS-induced NF-κB p65 expression, suggesting that RPTPβ/ζ may be involved in a novel mechanism of potentiation of microglial activation independent of the NF-κB p65 pathway. MY33-3 and MY10 limited LPS-induced nitrites production and *iNos* increases in BV2 microglial cells. SH-SY5Y neuronal cells were treated with the conditioned media from MY10/LPS-treated BV2 cells. Conditioned media from non-stimulated and from LPS-stimulated BV2 cells increased the viability of SH-SY5Y cultures. RPTPβ/ζ inhibition in microglial cells disrupted this neurotrophic effect of microglia, suggesting that RPTPβ/ζ plays a role in the neurotrophic phenotype of microglia and in microglia-neuron communication.

## Introduction

Neuroinflammation is an essential response mechanism to brain damage and activation of the central innate immune system is fundamental in the inflammatory process. Nevertheless, when unchecked, neuroinflammation has the potential to exert harmful effects on the integrity and function of the Central Nervous System (CNS)^1^. There are two main cell types that regulate neuroinflammation, namely astrocytes and microglia, the resident macrophages in the brain. Activated microglia can exert neurotrophic actions in the CNS by enhancing neurogenesis and suppressing neuroinflammation among other mechanisms^2^. However, chronic neuroinflammation, which is usually characterized by exacerbated and persistent microgliosis, has been largely associated with several pathologies such as multiple sclerosis^3^ or neurodegenerative diseases^4^. Accordingly, the cerebrospinal fluid of these patients shows elevated levels of pro-inflammatory factors such as IL-6, IL-1β and TNFα^5,6^. TNFα, for instance, is released by activated microglia and can induce cell death in models of neurodegeneration^7^. This dual role of neuroinflammation makes the identification of new modulatory mechanisms a pressing medical need.

In an effort to validate new targets in neuroinflammatory conditions, our strategy has been to identify molecules whose levels of expression are highly regulated in the brain in different pathologies with overt neuroinflammation^8^. Pleiotrophin (PTN) is an important cytokine for CNS repair and for survival and differentiation of neurons^9^. PTN expression is upregulated in the brain in different pathologies characterized by exacerbated neuroinflammatory processes such as Alzheimer’s disease, Parkinson’s disease (PD), ischemia, and after administration of drugs of abuse such as amphetamine and alcohol^8^. Using transgenic mice with PTN overexpression in the brain (*Ptn*-Tg) as a model of pathological disorders with upregulated PTN levels, we found that a single administration of lipopolysaccharide (LPS) induces an exacerbated microglial response in the area where the highest levels of expression of PTN are found, the prefrontal cortex (PFC)^10^.

Pleiotrophin signals through different receptors in different organs and contexts, being, potentially, Receptor Protein Tyrosine Phosphatase (RPTP) β/ζ the most relevant in the modulation of the effects of PTN in neuroinflammation^8^. PTN binds RPTPβ/ζ^11^, which is mainly expressed in the adult CNS in different cells including neurons and microglia^8,12^, and inactivates its phosphatase activity. This mechanism modulates the tyrosine phosphorylation of substrates of RPTPβ/ζ that are involved in neuroinflammation such as Anaplastic Lymphoma Kinase (ALK)^13^ and Fyn kinase^14^. LPS regulates inflammation through different pathways, some of which entail Fyn and ALK. LPS-induced activation of Toll Like Receptor 4 (TLR4) causes the phosphorylation and activation of Fyn. Activated Fyn triggers intracellular signalling pathways such as p38 MAPK and the subsequent increase in the transcription of different cytokines via NF-kB, including TNFα and IL-6^15^. IL-6 binds and activates its receptor, IL-6R, and induces the phosphorylation in tyrosine of signal transducer and activator of transcription-3 (STAT3)^16^, which is required for DNA binding and regulation of the expression of specific targets of proinflammatory genes such as TNFα or iNOS. Interestingly, ALK is a known regulator of the activation of STAT3^17^, suggesting a possible cross-talk between the PTN/RPTPβ/ζ and LPS signaling pathways. Despite this evidence, the possible modulatory role of RPTPβ/ζ on glial responses and neuroinflammation has not been studied yet.

In this work, we aimed to study the consequences of the modulation of the PTN/RPTPβ/ζ signaling pathway in microglial activation and neuroinflammation. For this purpose, we used genetic and pharmacological tools to regulate RPTPβ/ζ activity. First, we aimed to further clarify the effects of cerebral overexpression of PTN, endogenous inhibitor of RPTPβ/ζ, on LPS-induced glial responses and transcription of inflammatory mediators. Secondly, we tested the effects of selective small-molecule inhibitors of RPTPβ/ζ on LPS-induced glial responses and in primed microglia-neuron communication. The data presented here add to the knowledge of the modulatory actions of PTN in neuroinflammation and point to an important role of RPTPβ/ζ in neuroinflammation and the communication between microglia and neurons.

## Methods

### In vivo experiments

#### Animals

*Ptn*-Tg mice on a C57BL/6J background were generated by pronuclear injection as previously described^18,19^. PTN specific overexpression in different brain areas, including a ~ 2–3-fold upregulation in the PFC, was established by quantitative real time-polymerase chain reaction (qRT-PCR), in situ hybridization, and by Western blot^18,19^.

We used male *Ptn*-Tg and wild type (Wt) animals of 9–10 weeks (20–25 g). Mice were housed under controlled environmental conditions (22 ± 1 °C and a 12 h light/12 h dark cycle) with free access to food and water^10^.

All the animals used in this study were maintained in accordance with European Union Laboratory Animal Care Rules (86/609/ECC directive) and the protocols were approved by the Animal Research Committee of USP-CEU (authorization reference: PROEX 137/18).

#### Treatments

RPTPβ/ζ inhibitors (MY10 and MY33-3) were synthesized as previously described^20^.

To test genotypic differences in LPS-induced neuroinflammation in *Ptn*-Tg and Wt mice, we assessed the effects of a single IP injection of LPS (7.5 mg/kg) or saline (10 ml/kg) as a control. In the studies designed to test the effect of pharmacological inhibition of RPTPβ/ζ on LPS-induced neuroinflammation, Wt mice were administered 60 mg/kg MY10 or vehicle (10% dehydrated ethanol, 20% polysorbate 80, 70% PEG-400) by oral gavage in a volume of approximately 0.1 ml 1 h before a single IP injection of LPS (7.5 mg/kg) or saline (control). It was previously shown that the brain to plasma ratio is 3:1 1 h after intragastric administration of 60 mg/kg MY10^20^.

For immunohistochemistry analysis, animals were sacrificed 16 h after LPS or last saline administration (n = 4–5/group/genotype) by perfusion with 4% p-formaldehyde. For Western blot and qRT-PCR analysis, animals were decapitated 16 h after LPS or saline administration (n = 4–5/group/genotype) and PFC was rapidly removed and frozen in dry ice and stored at − 80 °C until the protein or RNA extraction procedures.

### Western blot

Protein extraction and quantification was performed as previously described^21^. Equilibrated protein samples were loaded onto 10% polyacrylamide gels, transferred to nitrocellulose membranes, probed with anti-phospho-STAT3 (1:1000) antibodies and re-probed with anti-STAT3 (1:1000) antibodies (Cell Signaling, Danvers, MA) and with anti-actin antibodies at a 1:5000 dilution (Chemicon, Temecula, CA). After incubation with appropriate secondary antibodies (1:5000) conjugated with horseradish peroxidase, the immunoreactive proteins were visualized using the ECL method according to the manufacturer’s instructions (Amersham, San Francisco, CA). Levels of phospho-STAT3 and STAT3 protein were quantified by densitometry in each animal sample using Image Lab image acquisition and analysis software (Bio-Rad, Hercules, CA).

### Immunohistochemistry

Thirty-micron PFC free-floating sections were processed as previously described^10,22^. Immunohistochemistry studies were performed in one slice per 180 µm (from bregma − 3.08 to − 2.46 mm).

Following previously published protocols^10^, sections were incubated overnight at 4 °C with anti-ionized calcium-binding adaptor molecule 1 (Iba1, Wako, Osaka, Japan; 1:1000), followed by 60 min incubation with the Alexa-Fluor-555 corresponding secondary antibody (Invitrogen, Waltham, MA USA; 1:500). For the study of p38 MAPK and NF-κB, antigen retrieval step was performed incubating sections in citrate buffer at 80 °C for 30 min. Sections incubated overnight at 4 °C with anti-glial fibrillary acidic protein (GFAP; Millipore, Madrid, Spain; 1:1000), anti-p38 MAPK and anti-NF-κB p65 antibodies (Cell Signaling, Danvers, MA) were then incubated for further 30 min with the corresponding biotinylated secondary antibodies, or Alexa-Fluor-488 corresponding secondary antibody (Invitrogen, Waltham, MA USA; 1:500) in PBS for NF-κB p65 antigen at room temperature. The avidin–biotin reaction was performed using a Vectastain Elite ABC peroxidase kit following the protocol suggested by the manufacturer. The immunoreactivity was visualized using 0.06% diaminobenzidine and 0.03% H_2_O_2_ diluted in PBS. Photomicrographs were captured with a digital camera coupled to an optical microscope (DM5500B) and the Leica SCN400 Scan Scanner (Leica, Solms, Germany). Analysis of microglia was performed in the three most central slices of PFC using ImageJ (NIH, Bethesda, MD, Version 1.50f) following methods previously used^10^. Iba1+ cells were counted in whole sections of PFC. Total marked area was calculated as overall image fluorescence, subtracting the mean background fluorescence. The "Analyze Particle" function in ImageJ software was used to study cell morphology. We measured the area and circularity of the objects that following the criteria previously reported^10^ met the minimum size to be analyzed. Cell area is expressed in square micrometers. Circularity was calculated as previously shown^10^: 4π × (area/perimeter^2^). Mean single cell values for each parameter were used for statistics.

### In vitro experiments

#### BV2 microglial cell cultures

BV2 murine microglial cells were a generous gift from Professor Antonio Cuadrado (Instituto de Investigaciones Biomédicas "Alberto Sols" (IIBM), Madrid, Spain). Cells were routinely maintained in RPMI-1640 medium with fetal bovine serum (10%), penicillin (100 U/ml), streptomycin (100 μg/ml) and L-glutamine (4 mM) at 37 °C in 5% CO_2_ humidified air following conditions previously reported^10^. Prior to each experiment, cells were grown for 24 h on 12-well plates at a concentration of 24 × 10^4^ cells per well to measure nitrites production or on 24-well plates at a concentration of 1 × 10^5^ cells to measure *iNos* and *Tnfα* mRNA levels (n = 4–6). After fasting cells for 24 h, BV2 cells were incubated with different concentrations of the RPTPβ/ζ inhibitors MY10 or MY33-3 (0.1, 1.0 and 10 µM) and with LPS (1 µg/ml) for another 24 h. For these cell treatments, RPTPβ/ζ inhibitors were dissolved in DMSO. Proper controls were added to discard the influence of 0.05% DMSO, the maximum concentration of DMSO used in the final in vitro experiments. All determinations were carried out in six replicates, and at least four independent experiments were performed.

#### Measurement of nitrites production

Nitrites accumulation in the BV2 culture medium was analyzed using the Griess reagent (2.25% sulfanilamide and 0.22% *N*-(1-naphthyl)-ethylenediamine dihydrochloride)^10^. Nitrites production was quantified in a microplate reader (Versa-Max, Molecular Devices, Sunnyvale, CA, USA) at 540 nm, and then calculated with reference to the standard curve generated with NaNO_2_. All determinations were carried out in six replicates, and at least four independent experiments were performed.

#### Neuroblastoma SH-SY5Y cells

SH-SY5Y cells were purchased from ATCC (Barcelona, Spain). Cells were routinely maintained in RPMI-1640 medium with fetal bovine serum (10%), penicillin (100 U/ml), streptomycin (100 μg/ml) and l-glutamine (4 mM) at 37 °C in 5% CO_2_ humidified air. Prior to each experiment, cells were grown for 24 h on 96-well plates at a concentration of 1 × 10^4^ cells per well. Cells were treated for 24 h with the conditioned media of BV2 cells that had been treated for 24 h with MY10 (0.1 μM, 1 μM and 10 μM) and/or LPS (1 μg/ml) as explained above. In control experiments, SH-SY5Y cells were directly treated with MY10 (0.1 μM, 1 μM and 10 μM) to assess possible effects of this compound on neuronal viability. Cell viability was assessed using the Methyl Thiazolyl Tetrazolium (MTT) assay^23^. Cells were incubated in a serum-free medium containing MTT solution in the dark for 4 h at 37 °C. The MTT solution was discarded and 100 μl DMSO was added to each well to dissolve the formazan crystals. The value of optical density was measured at a wavelength of 562 nm using a microplate reader (Versa-Max, Molecular Devices, Sunnyvale, CA, USA). Cell survival was calculated as a percentage relative to the control. All determinations were carried out in six replicates, and five independent experiments were performed.

### RNA extraction

Total RNA from PFC of Wt and *Ptn*-Tg mice, from BV2 cells and from SH-SY5Y cells was isolated with a commercial kit (Rneasy Mini kit, Qiagen, Valencia, CA, USA) after collecting samples in TRIzol reagent (Thermo Fisher Scientific, UK). The concentrations of RNA in each sample were measured at 260 nm and the integrity of RNA was confirmed in 1% agarose gels after electrophoresis.

### cDNA synthesis and SYBR green real-time quantitative-PCR analysis

First-strand cDNA of total RNA was synthesized using the iScript cDNA Synthesis kit (Bio-Rad Laboratories, Hercules, CA, USA). The cDNA was used for real-time quantitative PCR analysis using a CFX96 Real Time System (Bio-Rad Laboratories, Hercules, CA, USA). All reactions were performed in triplicate, in a total reaction volume of 15 μl.

The SYBR green RT-PCR method^24^ was performed using the following primer sets (forward and reverse):

For *Rpl13*: 5′GGTGCCCTACAGTTAGATACCAC3′ 5′ TTTGTTTCGCCTCCTTGGGTC3′; for *Hprt*: 5′TGCTCGAGATGTCATGAAGG3′ 5′TATGTCCCCCGTTGACTGAT3′; for *Tnfα*: 5′AGGCACTCCCCCAAAAGATG3′ 5′TGAGGGTCTGGGCCATAGAA3′; for *iNos*: 5′GGATGAGCTCATCTTTGCCACC3′ 5′GCATCTGGTAGCCAGCGTACC3′; for *Ptn*: 5′TTGGGGAGAATGTGACCTAATTAC3′ 5′GGCTTGGAGATGGTGACAGTTTTC3′; for *Casp3*: 5′TGGAACCAAAGATCATACATGGAA3′ 5′TTCCCTGAGGTTTGCTGCAT3′; for *Mfn2*: 5′CAGGACTGGTGGAGTCAACA3′ 5′AGAGCAGGGACATTGCGTTT3′; for *Edem*: 5′ACGAGCAGTGAAAGCCCTTTGG3′ 5′CCACTCTGCTTTCCAACCCAGT3′. The relative expression of each gene was normalized against *Hprt* and *Rpl13*, used as reference standard, as described by the manufacturer´s instructions of CFX96 Real Time System (Bio-Rad Laboratories, Hercules, CA, USA), and shown as fold change versus a control group.

### Statistics

Data are presented as mean ± standard error of the mean (S.E.M.). Data obtained from Western blot and qRT-PCR analysis of PFC from Wt and *Ptn*-Tg mice were analyzed using two-way ANOVA considering genotype and treatment as variants. Relevant differences were analyzed by post-hoc comparisons with Tukey’s post-hoc tests. The correlation between *Ptn* mRNA and the levels of *iNos* and *Tnfα* mRNA was analysed by Pearson correlation coefficient (r). Data obtained from Iba1 immunohistochemistry analysis and data obtained from cell cultures were analyzed using one-way ANOVA followed by post-hoc comparisons with Tukey’s post-hoc tests. The correlation between nitrites accumulation in the media from BV2 cells and SH-SY5Y cell viability was analysed by Pearson correlation coefficient (r). *P* < 0.05 was considered as statistically significant. All statistical analyses were performed using Graph-Pad Prism version 8 (San Diego, CA, USA).

## Results

### Correlation of pleiotrophin overexpression and proinflammatory markers in the PFC

**Figure 1:**
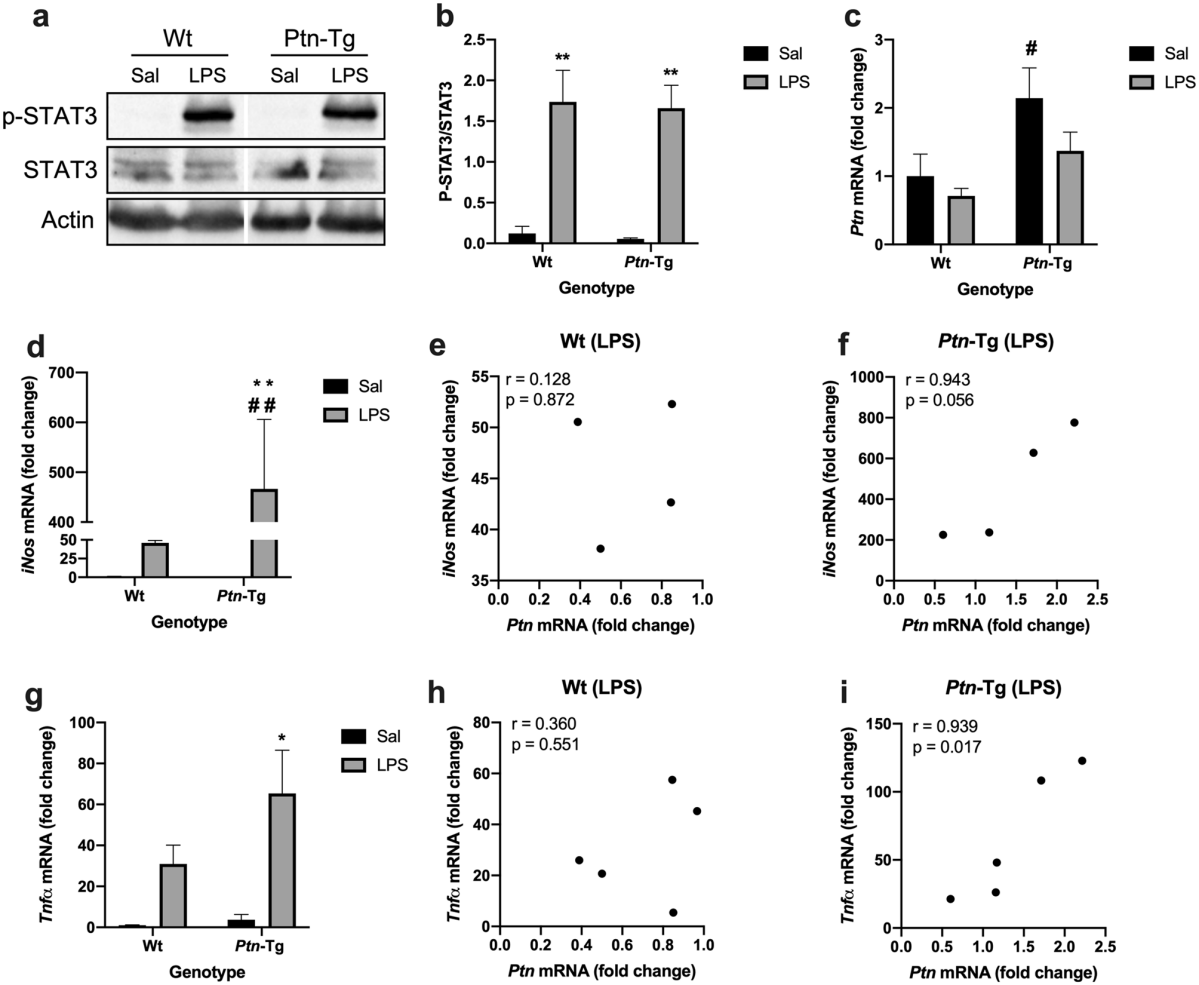
Effects of LPS on STAT3 phosphorylation and gene expression in the PFC of Wt and *Ptn*-Tg mice. Western blot assays using phospho-STAT3 and STAT3 antisera of PFC proteins extracts prepared 16 h after lipopolysaccharide (LPS) or saline (Sal) treatment (**a**). Optical density measurements did not reveal relevant differences between genotypes (**b**). Real-time PCR analyses of *Ptn* mRNA in PFC from Saline-treated (black bars) and LPS-treated (grey bars) Wt and *Ptn*-Tg mice (**c**). Real-time PCR analyses of *iNos* in PFC from Saline-treated and LPS-treated Wt and *Ptn*-Tg mice (**d**). Correlation between the mRNA of *iNos* and *Ptn* in PFC of LPS-treated Wt mice (**e**) and of LPS-treated *Ptn*-Tg mice (**f**). Real-time PCR analyses of *Tnfα* mRNA in PFC from Saline-treated and LPS-treated Wt and *Ptn*-Tg mice (**g**). Correlation between the mRNA of *Tnfα* and *Ptn* in PFC of LPS-treated Wt mice (**h**) and of LPS-treated *Ptn*-Tg mice (**i**). Pearson correlation coefficient (r) and p value are shown in all correlation graphs. Fold changes in the qPCR analysis are calculated relative to the control, Wt Sal. *P < 0.05 LPS vs. Sal. **P < 0.01 LPS vs. Sal. # *P* < 0.05 *Ptn*-Tg vs. Wt. ^##^*P* < 0.01 *Ptn*-Tg vs. Wt.

Previously, we showed that microgliosis is exacerbated in the PFC of *Ptn*-Tg mice treated with LPS, suggesting that PTN promotes LPS-induced neuroinflammation^10^. To shed some light on the mechanisms underlying the regulation of LPS effects by PTN, we studied the phosphorylation of STAT3, as a key event of the LPS/TLR4 signaling pathway, and proinflammatory markers in the same experimental paradigm: LPS (7.5 mg/kg)-injected *Ptn*-Tg mice. We found that LPS induced a robust phosphorylation of STAT3 in the PFC of Wt and *Ptn*-Tg mice 16 h after LPS administration (Fig. 1a,b, P = 0.0002); however, we did not observe differences between genotypes (Fig. 1a,b). Then, we measured *Ptn*, *iNos* and *Tnfα* mRNA levels in the PFC of these mice. As expected, ANOVA revealed a significant effect of the genotype in *Ptn* mRNA levels (F (1,16) = 8.38, *P* = 0.01). Saline-treated *Ptn*-Tg mice showed a significant upregulation of *Ptn* mRNA compared to Wt mice although this difference seemed to be attenuated 16 h after LPS treatment (Fig. 1c). We found a significant effect of the treatment (F (1,13) = 15.36, *P* = 0.002), of the genotype (F (1,13) = 10.35, *P* = 0.007) and of the interaction of both (F (1,13) = 10.44, *P* = 0.007) on *iNos* mRNA levels. We also found a significant effect of the treatment with LPS on *Tnfα* mRNA levels (F (1,14) = 12.21, *P* = 0.004). Both, *iNos* and *Tnfα* mRNA were upregulated in LPS-treated *Ptn*-Tg mice compared to Wt animals (Fig. 1d,g). We did not find a correlation between *iNos* or *Tnfα* mRNA and *Ptn* mRNA levels in the PFC of LPS-treated Wt mice (Fig. 1e,h). Interestingly, we found a significant correlation between the upregulated mRNA of *Ptn* in the PFC of *Ptn*-Tg mice treated with LPS and the expression of *iNos* and *Tnfα* mRNA (Fig. 1f,i).

### Inhibition of RPTPβ/ζ potentiates LPS-induced microglial response in vivo

**Figure 2:**
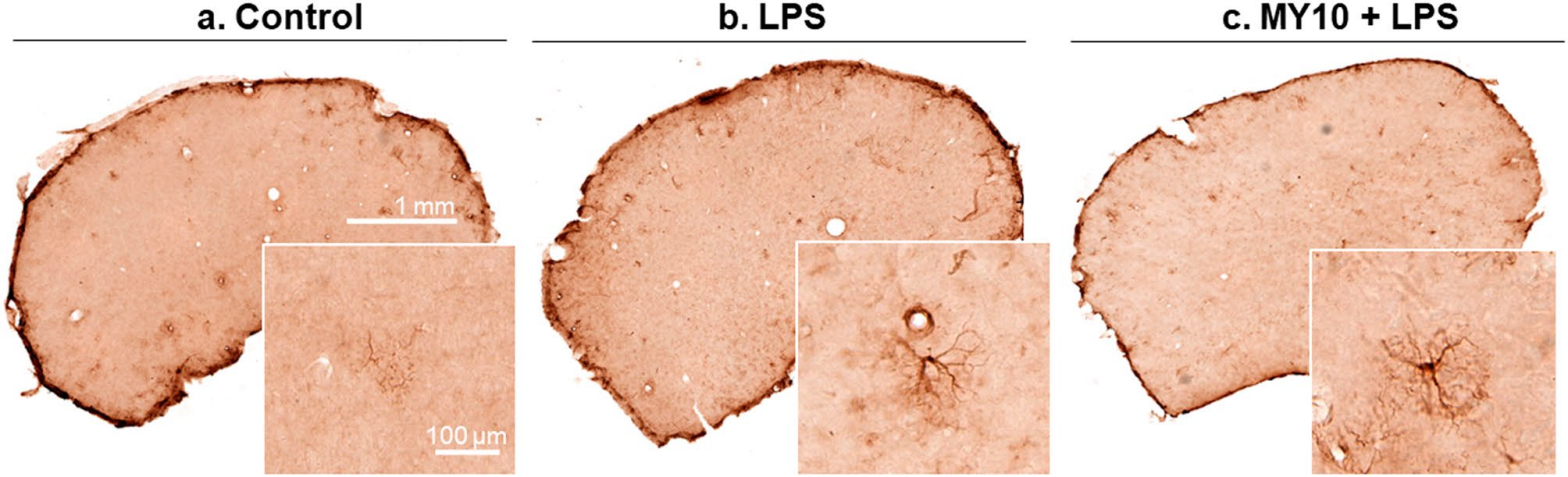
Effects of MY10 and LPS on astrocytosis in the mouse PFC. Photomicrographs are representative from GFAP-immunostained PFC sections of control (vehicle + saline) (**a**), LPS-treated (**b**) or MY10 + LPS-treated animals (**c**). Higher magnifications images in the lower right corner of every representative picture show hypertrophic and densely stained astrocytes in LPS-treated mice.

**Figure 3:**
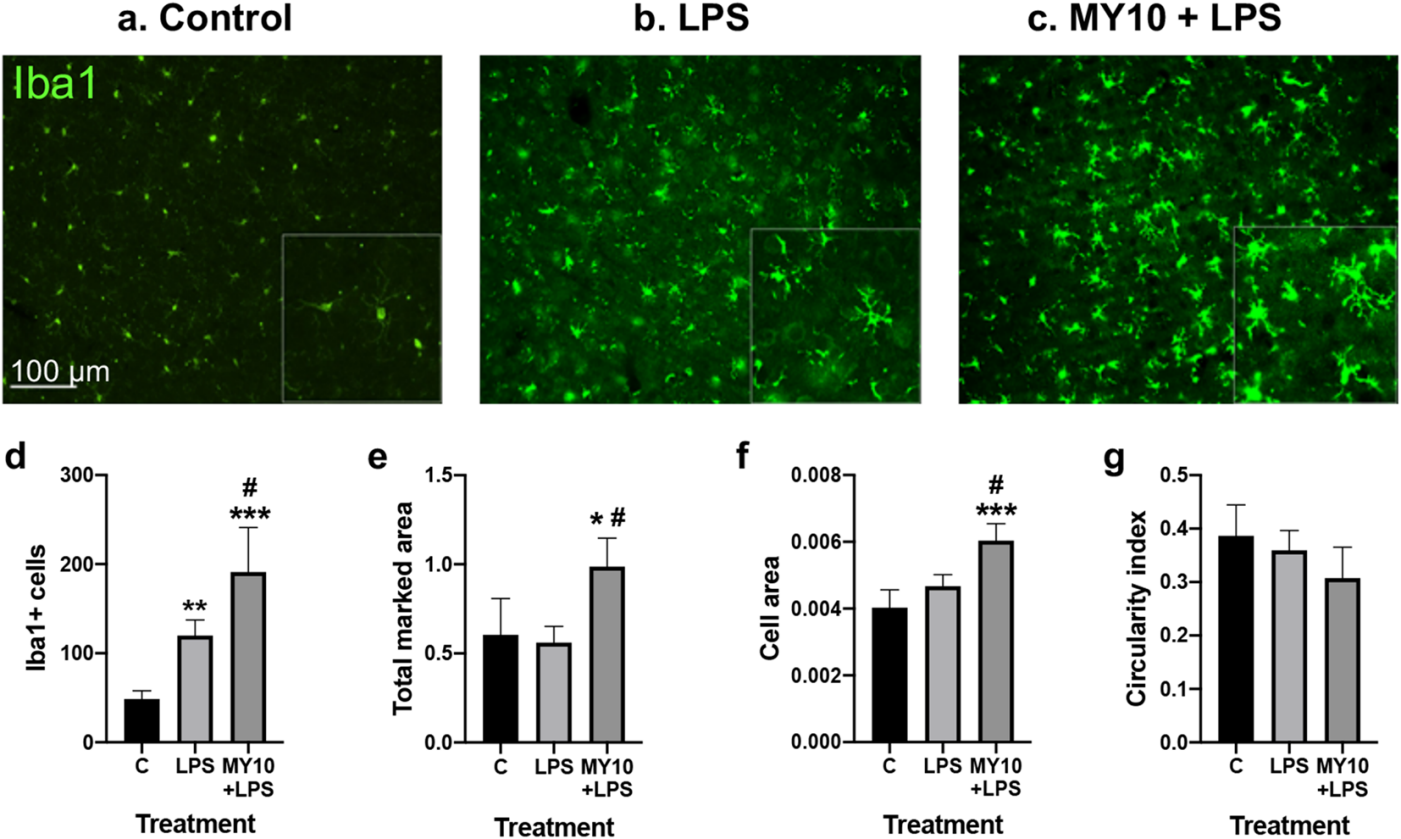
Effects of MY10 and LPS on microgliosis in the mouse PFC. Photomicrographs are representative from Iba1-immunostained PFC sections of control (vehicle + saline) (**a**), LPS-treated (**b**) or MY10 + LPS-treated animals (**c**). Higher magnifications images in the lower right corner of every representative picture show hypertrophic and densely stained microglia in LPS-treated mice. Graphs represent quantification of data (mean ± S.E.M) obtained from the counts of Iba-1-positive cells (**d**), total marked area (**e**), cell area (**f**) and circularity index (**g**). *P < 0.05; **P < 0.01; ***P < 0.001vs. control (C). ^#^*P* < 0.05 MY10 + LPS vs. LPS.

**Figure 4:**
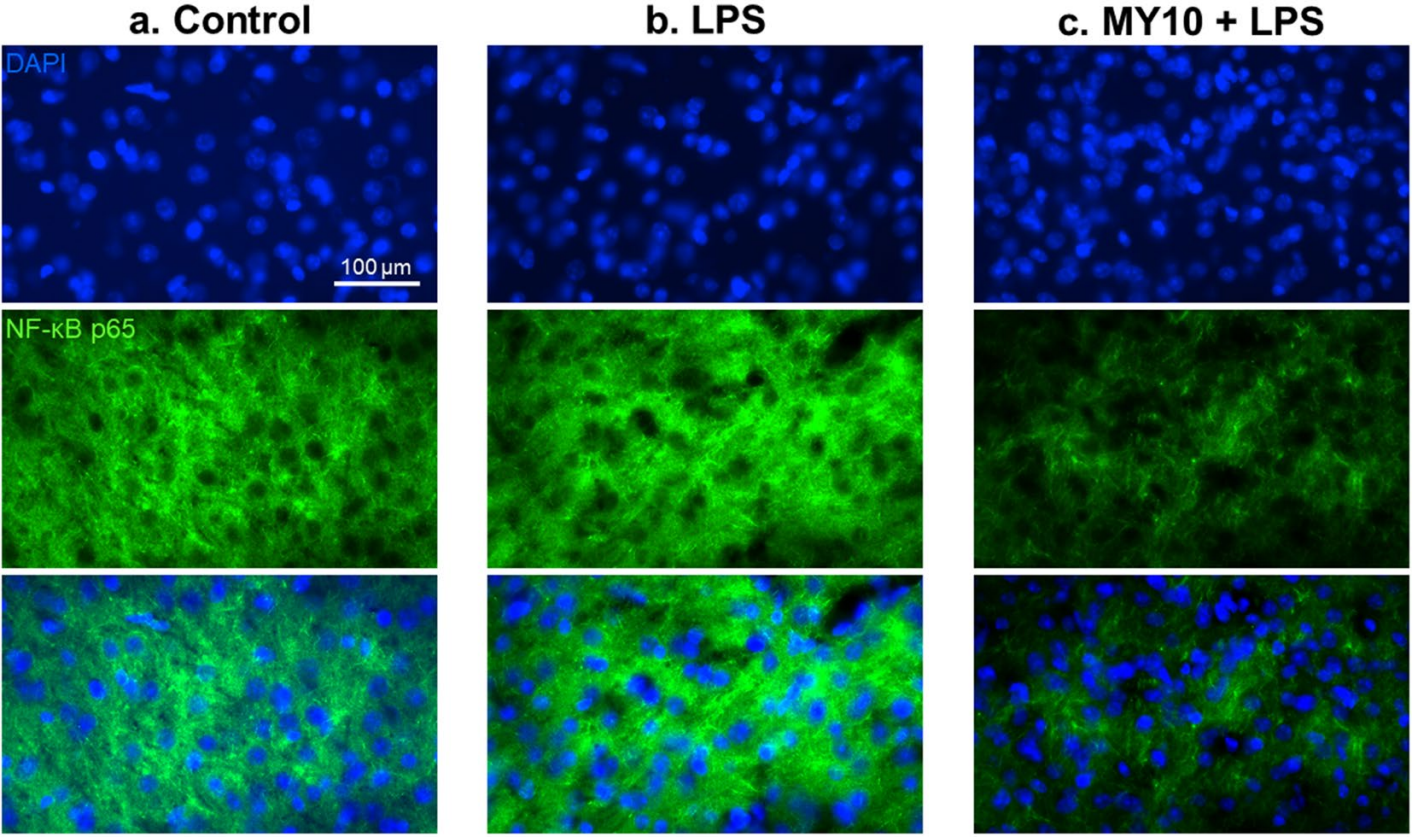
Effects of MY10 and LPS on NF-κB p65 expression in the mouse PFC. Photomicrographs are representative from DAPI- (blue) and NF-κB p65-immunostained (green) PFC sections of control (vehicle + saline) (**a**), LPS-treated (**b**) or MY10 + LPS-treated animals (**c**).

To test the possibility that the PTN receptor RPTPβ/ζ may be involved in the regulation of LPS effects in vivo, we used the same LPS treatment protocol in Wt mice pretreated with the RPTPβ/ζ inhibitor MY10. In immunohistochemistry studies, we tested a possible modulatory role of RPTPβ/ζ on LPS-induced gliosis. LPS induced an astrocytic response in the PFC compared with control animals (Fig. 2a,b), whereas MY10 alone did not show relevant effects (data not shown). Treatment with MY10 did not show important effects on LPS-induced astrocytosis (Fig. 2c). In contrast, relevant differences were found when we analyzed the regulation of LPS-induced microglial response. We observed that treatment with MY10 potentiated LPS-induced microgliosis compared with mice treated only with LPS (Fig. 3a–c). Accordingly, the number of Iba1+ cells was significantly affected by the treatment (F (2,9) = 27.98, *P* = 0.0001). LPS treatment induced an increase in the number of Iba1+ cells (Fig. 3d) that was significantly enhanced by concomitant treatment with MY10 (Fig. 3d). Treatment also showed significant effects on total marked area (F (2, 9) = 6.84, *P* = 0.01) and cell area (F (2, 9) = 17.12, *P* = 0.0009). Although these parameters did not seem to be affected by treatment with LPS, they were significantly increased by treatment with MY10 and LPS (Fig. 3e,f). Finally, although we did not detect significant effects of the treatments on circularity (F (2, 9) = 2.164, *P* = 0.17), we observed that treatments with LPS and MY10 with LPS tended to decrease the circularity of these cells (Fig. 3g). Overall, the data demonstrate that LPS-induced microglial response in the PFC is increased by treatment with MY10, suggesting a role of RPTPβ/ζ on LPS-induced microglial activation. As it happened in the studies with Ptn-Tg mice (Fig. 1a,b), we did not observe modulatory effects of MY10 on LPS-induced phosphorylation of STAT-3 in Wt mice (data not shown). To further study the possible modulation by MY10 of other molecules involved in the process of neuroinflammation, we tested the expression of p38 MAPK and NF-κB p65 in the PFC of Wt mice treated with MY10 and LPS. We did not find any treatment-related differences in the pattern of expression of p38 in the mouse PFC (Supplementary Fig. 1). Mice treated with LPS exhibited a moderate increase of NF-κB p65 protein expression in the PFC (Fig. 4a,b). However, we observed that treatment with MY10 caused a clear decrease in LPS-induced NF-κB p65 protein expression (Fig. 4c). NF-κB p65 nuclear staining was only residually observed, independently of the treatment considered. Overall, the data suggest that potentiation of LPS-induced microgliosis by MY10 may occur through signaling independent of the prototypical NF-κB p50/p65 transcription factor.

### Modulatory role of RPTPβ/ζ on microglial activation in vitro

**Figure 5:**
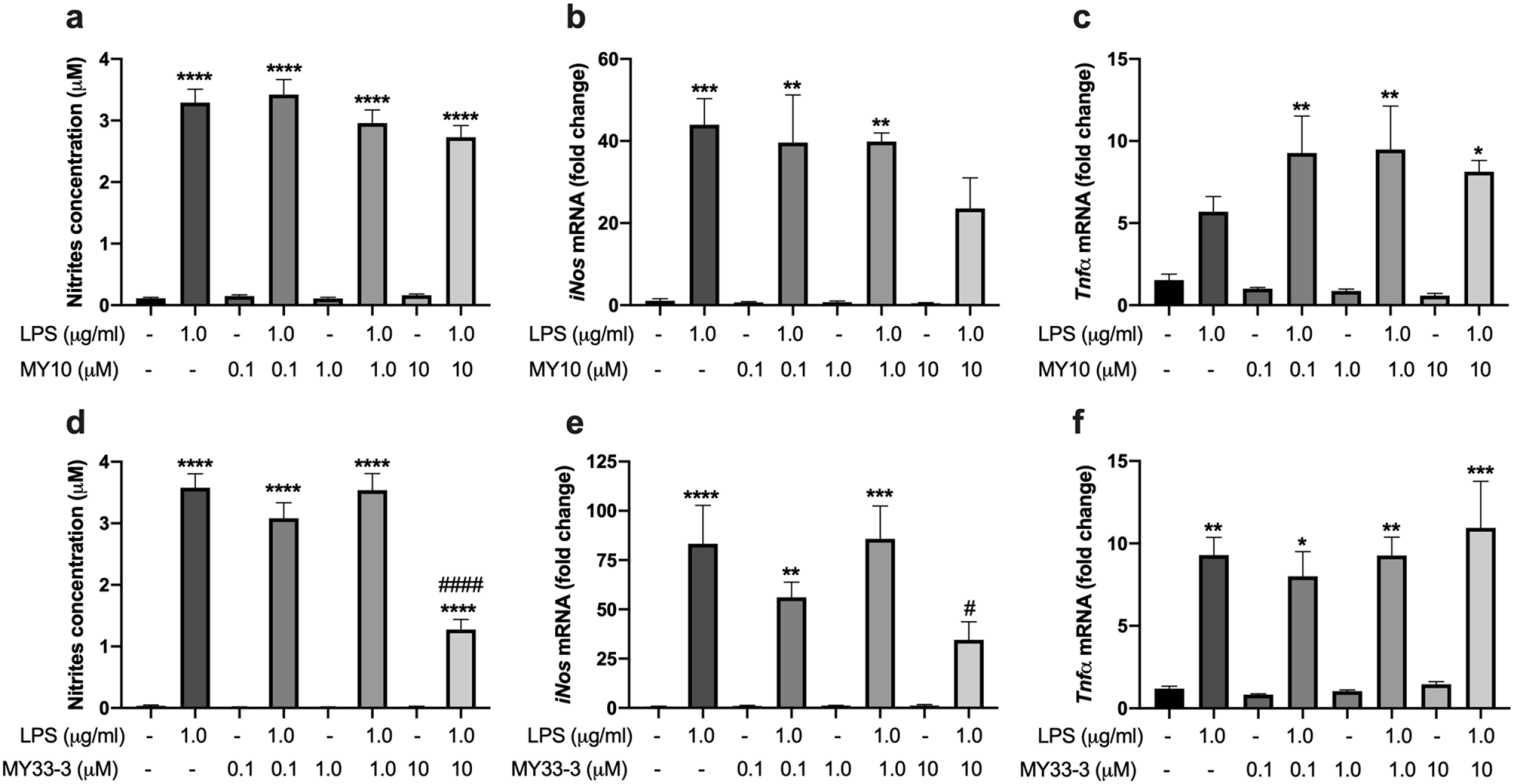
Effects of inhibition of RPTPβ/ζ on LPS-induced nitrites production and in *iNos* and *Tnfα* mRNA levels in BV2 microglial cells. Cells were treated with the indicated concentrations of MY10 or MY33-3 (0.1, 1.0 or 10 μM) and/or with LPS (1.0 μg/ml) for 24 h. Nitrites accumulation in the BV2 culture medium was analyzed using the Griess reactive (**a**,**d**). Real-time PCR analyses of *iNos* (**b**,**e**) and *Tnfα* mRNA (**c**,**f**). *P < 0.05; **P < 0.01; ***P < 0.001; ****P < 0.0001 vs. control non-stimulated cells (without LPS and MY10 or MY33-3, black bar). ^#^*P* < 0.05; ^####^*P* < 0.0001 vs. LPS-stimulated cells (without MY10 or MY33-3, dark grey bar).

To test the effect of the inhibition of RPTPβ/ζ on LPS effects in microglial cells, we stimulated BV2 microglial cells with MY10 and LPS, and measured nitrites concentration in the media as a correlate of microglial activation. As expected, LPS induced a highly significant increase of nitrites production by BV2 cells (Fig. 5a; *P* < 0.0001). Coincubation with the highest concentration of MY10 (10 μM) tended to limit LPS-induced nitrites production but no significant effects were detected (Fig. 5a). Accordingly, LPS induced a significant increase of *iNos* mRNA in BV2 cells (Fig. 5b; *P* = 0.0005); however, LPS-induced increase of *iNos* mRNA levels in BV2 cells was not statistically significant when cells were co-treated with LPS and 10 μM MY10 (Fig. 5b). In contrast, MY10 tended to potentiate LPS-induced increase of *Tnfα* mRNA in BV2 cells (Fig. 5c). To confirm the relevance of the modulatory effects of RPTPβ/ζ inhibition on LPS actions in microglial cells, we tested another inhibitor of RPTPβ/ζ: MY33-3^20^. The highest concentration of MY33-3 (10 μM) significantly limited both the LPS-induced nitrites production (Fig. 5d; *P* < 0.0001) and the increase of *iNos* mRNA (Fig. 5e; *P* = 0.03). Overall, the data suggest direct modulatory actions of RPTPβ/ζ on microglial activation. In the case of *Tnfα*, MY33-3 did not seem to exert any effect on the mRNA levels of this cytokine after LPS stimulation (Fig. 5f). The apparent discrepancy with the effects observed with MY10 (Fig. 5c) might only reflect that in the experiments carried out with MY10, the effect of LPS alone on *Tnfα* mRNA was smaller than in the experiments performed with MY33-3 (Fig. 5c,f).

### Modulatory role of RPTPβ/ζ in microglia-neuron communication

**Figure 6:**
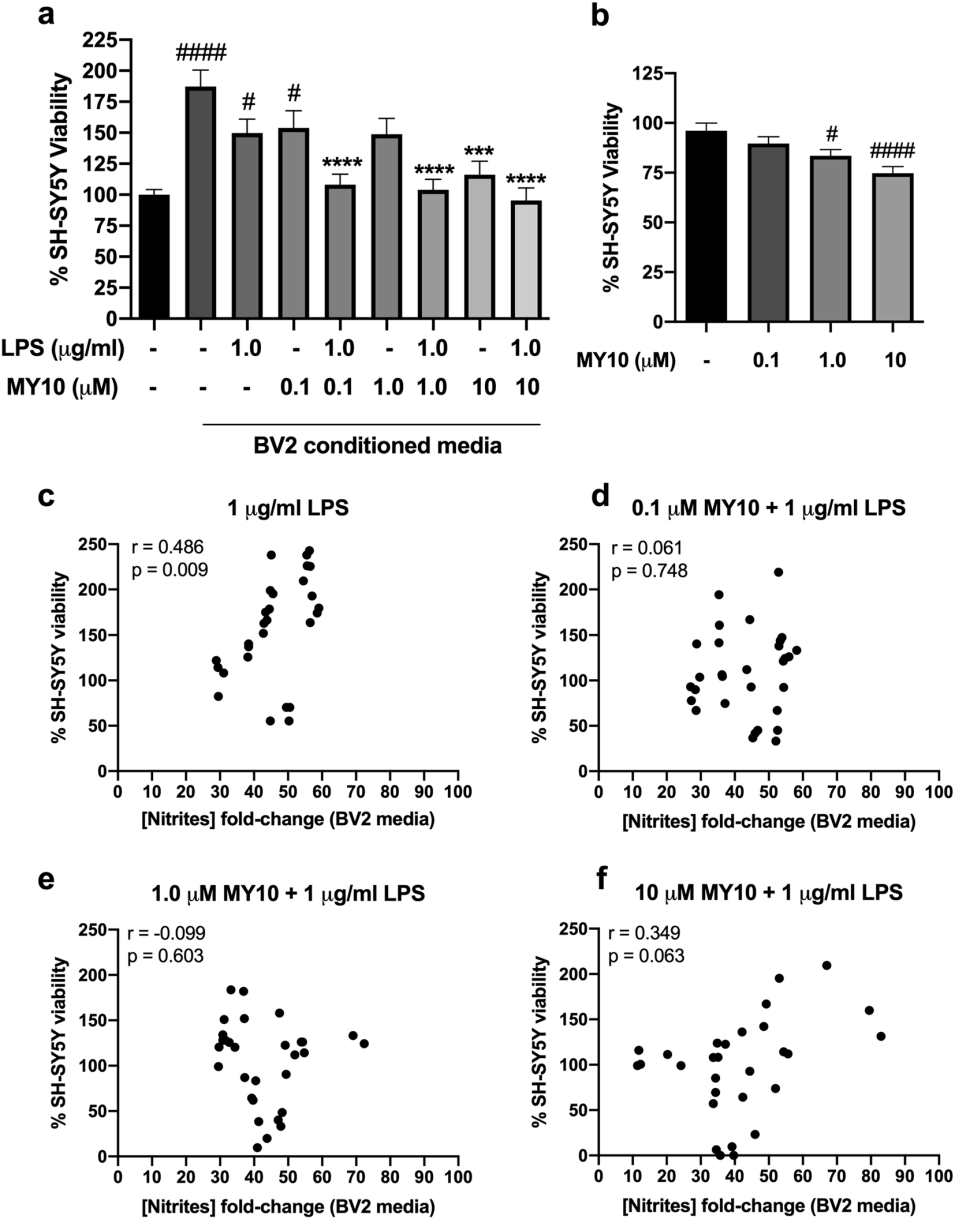
Pharmacological inhibition of RPTPβ/ζ prevents the neurotrophic phenotype of microglia. MY10 significantly decreases the neurotrophic effects of media from BV2 cells when SH-SY5Y neurons are incubated with the media from MY10/LPS-treated BV2 cells, measured by the MTT test (**a**). High concentrations of MY10 decrease the viability of SH-SY5Y neurons in the MTT test (**b**). Correlation between the nitrites production of LPS-treated BV2 cells and SH-SY5Y cell viability (**c**). Correlation between the SH-SY5Y cell viability and the nitrites production of 0.1 μM MY10 + LPS-treated BV2 cells (**d**), 1 μM MY10 + LPS-treated BV2 cells (**e**) and 10 μM MY10 + LPS-treated BV2 cells (**f**). Pearson correlation coefficients (r) and p values are shown in all correlation graphs. ***P < 0.001; ****P < 0.0001 vs. non-stimulated BV2 cells conditioned media (without LPS and MY10; dark grey bar). ^#^*P* < 0.05; ^####^*P* < 0.0001 vs. control SH-SY5Y cell just incubated with control media (black bar). Data are shown as % viability considering 100% viability of neurons incubated with control media (black bar).

Since microglial activation and release of different mediators exert diverse effects on neurons, we wanted to test whether some of these effects depend on the activity of RPTPβ/ζ in microglial cells. For this purpose, we tested the effects of the conditioned media from MY10/LPS-treated BV2 microglial cells on the viability of SH-SY5Y neuronal cultures. First, we found that conditioned media from fasted non-stimulated BV2 cells significantly increased the viability of SH-SY5Y cultures compared to control SH-SY5Y cells (Fig. 6a; *P* < 0.0001), suggesting the release from resting BV2 microglial cells of neurotrophic factors for SH-SY5Y cells. This effect was less evident when SH-SY5Y cells were incubated for 24 h with the conditioned media from LPS-stimulated BV2 cells but could still be observed (Fig. 6a; *P* = 0.04). Interestingly, this limiting effect of LPS on the neurotrophic actions of the media from BV2 cells was significantly potentiated by MY10 (Fig. 6a). Surprisingly, the highest concentration of MY10 alone (10 μM) also limited the neurotrophic actions of BV2 media on SH-SY5Y cells (Fig. 6a; *P* = 0.0002), suggesting that RPTPβ/ζ is a relevant factor for neuroprotective microglia. As a control of the possible effects of MY10 remnants in the conditioned media from BV2 cells, we incubated SH-SY5Y cells with the same concentrations of MY10. We did not observe a significant effect of the lowest concentration of MY10 (0.1 μM); however, we observed a significant ~ 20–25% reduction of the viability of cells incubated with 1 μM MY10 (Fig. 6b; *P* = 0.04) and 10 μM MY10 (Fig. 6b; *P* < 0.0001). The data suggest that RPTPβ/ζ activity is necessary for healthy SH-SY5Y cultures. Thus, we aimed to test if the MY10-induced reduction of the increase of the viability of SH-SY5Y cultures treated with conditioned media from fasted non-stimulated BV2 cells could be related to effects of MY10 on apoptosis, endoplasmic reticulum stress or changes in mitochondrial function. For this purpose, we measured the mRNA levels of *Casp3* (caspase 3), *Edem* (ERAD-enhancing α-mannosidase-like protein) and *Mfn2* (mitofusin 2) in SH-SY5Y cells treated with the conditioned media of BV2 cells. We did not find any treatment-related changes in the levels of expression of these genes (Supplementary Fig. 2).

Finally, since there is some controversy about the possible neurotrophic effects of acute microglial activation, we tested the possible correlation between the nitrites production of LPS-stimulated BV2 cells and the viability of SH-SY5Y neurons (Fig. 6c–f). We found a positive correlation between nitrites production on LPS-stimulated BV2 cells and viability of SH-SY5Y cells incubated with the media from those BV2 cells (Fig. 6c). Very interestingly, that correlation is lost when BV2 cells were stimulated with LPS and different concentrations of MY10 (Fig. 6d,f). The data suggest that RPTPβ/ζ activity may be involved in the neuroprotective mechanisms triggered by primed microglia.

## Discussion

Using genetic mouse models of *Ptn* overexpression or deletion^10,22^, we previously showed that PTN regulates the astrocytic and microglial responses after LPS treatment. To further clarify the role of PTN in neuroinflammation, we have now used the same model of LPS-treated *Ptn*-Tg mice to assess the possibility that PTN overexpression in the brain modulates LPS signaling. The data demonstrate that the enhanced microglial response induced by LPS in the PFC of *Ptn*-Tg mice^10^ is not related to changes in the activation of STAT3 after LPS stimulation. We also studied the putative correlation between individual *Ptn* mRNA in the PFC and the mRNA of inflammatory markers after LPS administration. The upregulation of *Ptn* mRNA in the PFC of saline-treated *Ptn*-Tg mice compared to Wt animals seemed to be attenuated after LPS treatment. However, we have to consider that these mRNA levels are measured 16 h after treatment with LPS. It is reasonable to think that the actual upregulation of *Ptn* in the moment of LPS injection in those animals, which is assumed to be similar to that found in saline-treated mice, had also an impact in the mouse response to LPS treatment. We found a positive correlation between the upregulated levels of *Ptn* expression and LPS-induced increases of *iNos* and *Tnfα* only in the PFC of *Ptn*-Tg mice, not in Wt mice. These results are important since there is a number of brain related pathologies characterized by upregulation of PTN^8^, suggesting that this cytokine is relevant for the neuroinflammatory processes described as a hallmark of those disorders.

**Figure 7:**
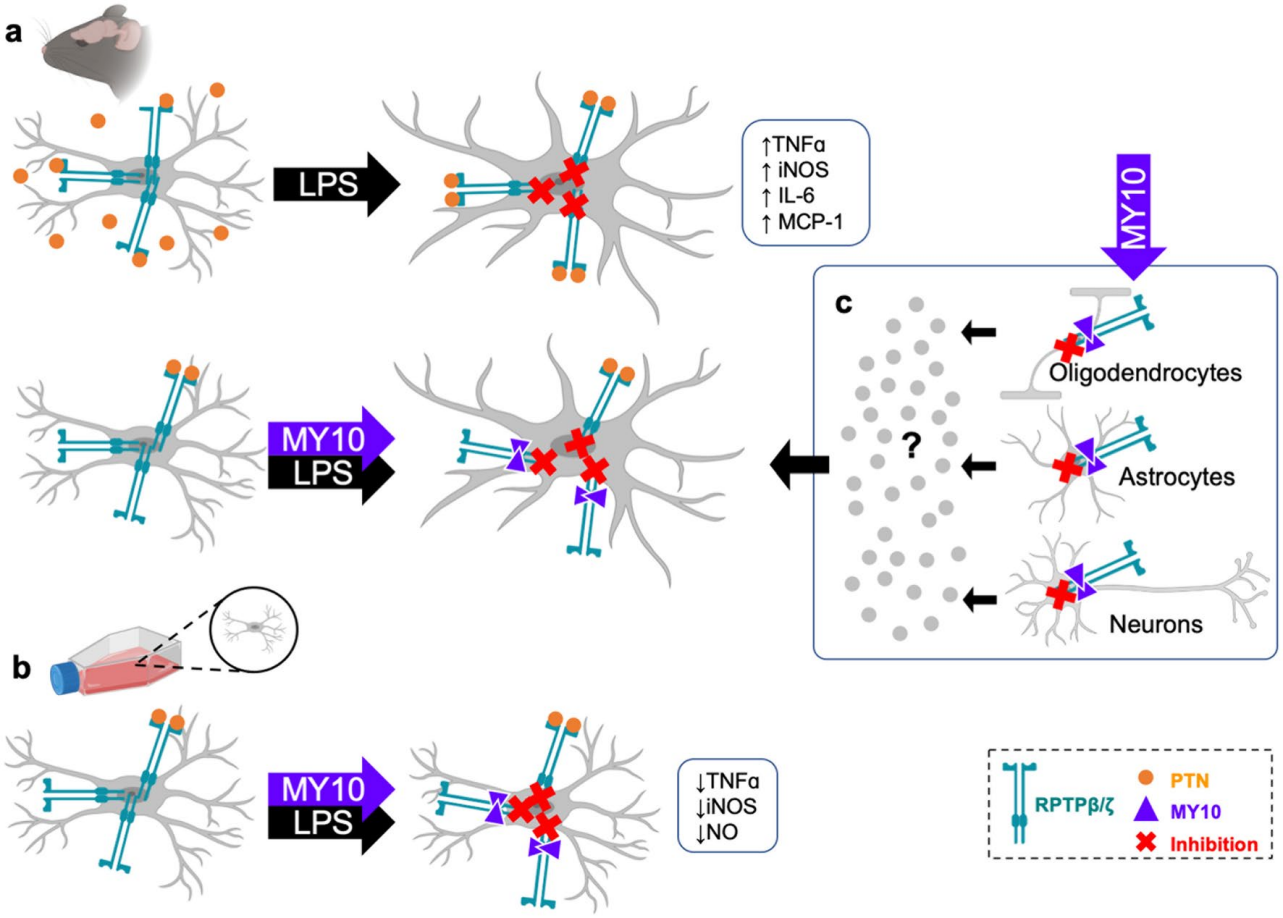
Inhibition of RPTPβ/ζ differentially modulates LPS-induced microglial responses in vivo and in vitro. Transgenic overexpression of the endogenous RPTPβ/ζ inhibitor PTN in the mouse brain potentiates LPS-induced microgliosis and LPS-induced increases of inflammatory mediators^10^ (**a**). Accordingly, we now demonstrate that systemic administration of the exogenous RPTPβ/ζ inhibitor MY10 potentiates LPS-induced microgliosis in the mouse PFC. In contrast, when acting in isolated microglial cells in vitro*,* MY10 limits the LPS-induced production of inflammatory mediators such as TNFα, iNOS and NO (**b**). We hypothesize that the overall potentiation of LPS-induced microgliosis by MY10 in vivo may reflect the actions of the inhibition of RPTPβ/ζ in different cells in the brain. RPTPβ/ζ is also expressed in oligodendrocytes, astrocytes and neurons. Inhibition of the phosphatase activity of RPTPβ/ζ in these cells could alter their communication with microglia (e.g. through changes in the secretome of these cells), potentially impacting microglial cells in different ways (**c**). This figure was created with BioRender's web-based software—https://biorender.com/.

To shed light on the molecular mechanisms mediating the PTN effects on glial responses and neuroinflammation, we decided to study the role of its receptor, RPTPβ/ζ, in these processes. Using a small-molecule inhibitor of this receptor (MY10), we demonstrate here for the first time that pharmacological inhibition of RPTPβ/ζ potentiates LPS-induced microgliosis in the mouse PFC. This result is in agreement with previous studies showing that overexpression of PTN, the endogenous inhibitor of RPTPβ/ζ, causes similar effects in mice^10^ (Fig. 7). A known substrate of RPTPβ/ζ, ALK^13^, regulates the activation of STAT3 through modulation of its tyrosine phosphorylation^17^ and has been linked to the activation of the inflammasome (NLRP3) in macrophages, which triggers a rapid immune response against pathogen-associated molecular patterns (PAMPs)^25^. However, as it happened in *Ptn*-Tg mice, treatment with MY10 did not seem to regulate the phosphorylation of STAT3 in the PFC of mice treated with LPS, suggesting that the regulation of LPS-induced microglial response by MY10 is independent of STAT3. Then, we tested the possibility that MY10 modulates LPS-induced NF-κB p65 expression. Functional NF-κB p65 participates in combination with other transcription factors in activation of pro-inflammatory genes, making NF-κB p65 the most likely candidate for initiation and amplification of neuroinflammation. Treatment with MY10 1 h before LPS caused a decrease in NF-κB p65 expression, which was only residually detected in nuclei of all groups. Taken together, the predominant extranuclear localization of NF-κB p65 and the absence of correlation between MY10 potentiation of LPS-induced microglial priming in the PFC and prototypical pro-inflammatory signaling, the data suggest the existence of an unknown promoter of microgliosis. This circumstance of potentiation of microgliosis together with the decrease of NF-κB p65 has been described in other contexts such as aging^26^ and point to a novel RPTPβ/ζ mediated signaling mechanism of potentiation of LPS-induced microglial activation, which is independent of the NF-κB p65 pathway. However, a limitation of this study has to be noted. These experiments were carried out 16 h after a single LPS administration, a valuable time point to study glial responses^10^. However, NF-κB p65 nuclear expression has been shown in vivo after repeated LPS administrations, or shortly after a single LPS administration with the resulting increase of serum TNFα levels returning to basal 9 h after the administration of the toxin^27,28^.

The limitation of solely testing the effects of LPS 16 h after the administration of the toxin, may also underlie the absence of influential effects of MY10 on the modulation of LPS-induced astrocytic response. It should be noted that astrocytic activation is usually more evident following microglial activation, since the astrocytic response is enhanced by proinflammatory cytokines secreted by microglia in response to the inflammatory stimulus^29^.

Since RPTPβ/ζ is known to be expressed in microglia^30,31^, we hypothesized that direct inhibition of RPTPβ/ζ in microglial cells could regulate LPS-induced microglial responses. Our data confirmed this hypothesis but, surprisingly, we found that inhibition of RPTPβ/ζ in microglial cells using different inhibitors, MY10 and MY33-3, decreases LPS-induced nitrites production and LPS-induced upregulation of *iNos* mRNA (Fig. 7). This apparent discrepancy could be explained by the ability of PTN to bind different receptors including syndecan-3 and ALK^8^. Although the pattern of expression of RPTPβ/ζ in the brain makes it a probable primary target for PTN in the CNS, we cannot rule out the possibility of the contribution of other receptors mediating PTN actions. Also, taking together the in vivo and in vitro evidence presented here, the data suggest that the overall potentiation of LPS-induced microgliosis by MY10 in vivo may reflect the actions of the inhibition of RPTPβ/ζ in different cells in the brain. RPTPβ/ζ is also expressed in oligodendrocytes, astrocytes and neurons^32^ and inhibition of its phosphatase activity by MY10 could cause changes in the secretome of those cells, potentially impacting microglial cells in different ways (Fig. 7). In fact, a role of RPTPβ/ζ in the modulation of the contents of exosomes of different cells, such as adipocytes, has been proposed^8^.

The direct effects of RPTPβ/ζ inhibition in BV2 microglial cells were intriguing. As mentioned before, PTN, the endogenous inhibitor of RPTPβ/ζ, is a well-established neurotrophic factor and a modulator of microglial responses. However, it was not known whether RPTPβ/ζ inhibition in microglial cells is a signaling event driving these cells to a neuroprotective or deleterious phenotype. To answer this question, we stimulated the neuronal cell line SH-SY5Y with the conditioned media from BV2 cells incubated with LPS and MY10. Interestingly, we observed that fasting media from non-stimulated BV2 cells induced a significant increase in the viability of SH-SY5Y cell cultures. Despite the MTT test does not discriminate between effects on viability or proliferative effects, the data suggest neurotrophic effects of resting microglia. The concept of resting microglia acquiring different phenotypes to contribute to CNS homeostasis was already known^33^. Both resting and primed microglia are able to produce a number of cytokines, prostaglandins and neurotrophic factors, such as Brain-Derived Neurotrophic Factor (BDNF), that influence and modulate neuronal function and survival. Moreover, release of extracellular vesicles (EVs) from microglia allows the exchange of a wide variety of biomolecules with neurons^34^.

Currently, there is a pressing need to dissect the modulatory mechanisms of microglia-neuron communication in health and disease. In our studies, it was interesting to see that treatment with the highest concentration of MY10 alone prevented the neurotrophic effect of the media from BV2 microglial cells. These data needed to be considered with caution because in control studies we observed that the highest concentrations of MY10 decrease the viability of SH-SY5Y cells. However, our data suggest that the limitation of the neurotrophic effects of the conditioned media of BV2 cells treated with MY10 or MY10 and LPS is not related to apoptotic effects, endoplasmic reticulum stress or dysregulation of mitochondrial function of SH-SY5Y cells. It does not seem related either to the capacity of MY10 to modulate microglial activation because the incubation of BV2 cells with MY10 alone did not alter nitrites production nor the levels of *iNos* or *Tnfα*. It was also interesting to observe that the neurotrophic effects of resting microglia were slightly attenuated in LPS-primed microglia. More importantly, the combination of MY10 and LPS fully prevented this neurotrophic phenotype of microglia. Accordingly, the positive correlation between viability of neuronal cells and the nitrites production stimulated by LPS in microglial cells is lost when BV2 cells were simultaneously treated with LPS and the RPTPβ/ζ inhibitor MY10. Overall, the data strongly suggest the possibility that RPTPβ/ζ inhibition in microglial cells disrupts the neuroprotective phenotype of microglia.

In summary, the data demonstrate that upregulated levels of PTN correlate with inflammatory markers in the PFC of LPS-treated mice. The receptor of PTN, RPTPβ/ζ, is involved in a novel mechanism of potentiation of microglial activation in vivo that seems to be independent of the NF-κB p65 pathway. The data suggest an important role of RPTPβ/ζ in the neurotrophic actions of resting and primed microglia, and in the microglia-neuron communication.

## Supplementary information

Supplementary Figures.

## Data Availability

The datasets used and/or analysed during the current study available from the corresponding author on reasonable request.
