# Supplementary material for: Role of RPTPβ/ζ in neuroinflammation and microglia-neuron communication
Source: Sci Rep. 2020 Nov 20;10:20259. doi: 10.1038/s41598-020-76415-5 (PMC7679445; doi:10.1038/s41598-020-76415-5)
Supplement: Supplementary file 1 — Supplementary Figures. [file 41598_2020_76415_MOESM1_ESM.docx]

**Role of RPTPβ/ζ in neuroinflammation and microglia-neuron communication**

Rosalía Fernández-Calle ^1^, Milagros Galán-Llario ^1^, Esther Gramage ^1^, Begoña Zapatería ^2^, Marta Vicente-Rodríguez ^1^, José M. Zapico ^2^, Beatriz de Pascual-Teresa ^2^, Ana Ramos ^2^, M. Pilar Ramos-Álvarez ^2^, María Uribarri ^3^, Marcel Ferrer-Alcón ^3^, Gonzalo Herradón ^1,*^

^1^ Departamento de Ciencias Farmacéuticas y de la Salud, Facultad de Farmacia, Universidad San Pablo-CEU, CEU Universities, Urbanización Montepríncipe, 28925, Alcorcón, Madrid, Spain

^2^ Departamento de Química y Bioquímica, Facultad de Farmacia, Universidad San Pablo-CEU, CEU Universities, Urbanización Montepríncipe, 28925, Alcorcón, Madrid, Spain

^3^ BRAINco Biopharma, S.L., Bizkaia Technology Park, Spain

*Corresponding author:

Gonzalo Herradón, PhD

Lab. Pharmacology

Facultad de Farmacia

Universidad San Pablo-CEU

Urb. Montepríncipe

28925, Alcorcón, Madrid, Spain.

Tel: 34-91-3724700 (Ext. 14840)

Fax: 34-91-3510475

e-mail: herradon@ceu.es

**Supplementary Figure 1.**

**
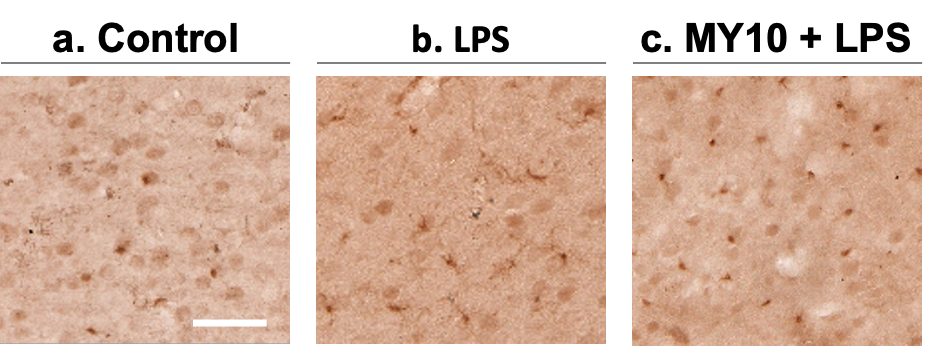
**

**Effects of MY10 and LPS on p38 MAPK in the mouse PFC.** Photomicrographs are representative from p38-immunostained PFC sections of control (vehicle + saline) (a), LPS-treated (b) or MY10 + LPS-treated animals (c). Scale bar = 100 μm.

**Supplementary Figure 2.**

**Effects of conditioned media from BV2 microglial cells treated with MY10 and/or LPS in *Casp3, Edem* and *Mfn2* mRNA levels in SH-SY5Y neuronal cells.** BV2 cells were treated with the indicated concentrations of MY10 (0.1, 1.0 or 10 μM) and/or with LPS (1.0 μg/ml) for 24 h. SH-SY5Y cells were treated with conditioned media from those BV2 cells for 24 h. Real-time PCR analyses of *Casp3, Edem* and *Mfn2* mRNA in SH-SY5Y cells are represented.

**Supplementary Figure 3.**

Blots from Fig. 1a.
